# Prevalence of Virus Infections and GLRaV-3 Genetic Diversity in Selected Clones of Croatian Indigenous Grapevine Cultivar Plavac Mali

**DOI:** 10.3390/pathogens11020176

**Published:** 2022-01-27

**Authors:** Mate Čarija, Tomislav Radić, Silvija Černi, Ana Mucalo, Goran Zdunić, Darko Vončina, Martin Jagunić, Katarina Hančević

**Affiliations:** 1 Institute for Adriatic Crops, 21000 Split, Croatia; mate.carija@krs.hr (M.Č.); tomislav.radic@krs.hr (T.R.); ana.mucalo@krs.hr (A.M.); goran.zdunic@krs.hr (G.Z.); 2 Department of Biology, Faculty of Science, University of Zagreb, 10000 Zagreb, Croatia; silvija.cerni@biol.pmf.hr; 3 Department of Plant Pathology, Faculty of Agriculture, University of Zagreb, 10000 Zagreb, Croatia; dvoncina@agr.hr (D.V.); mjagunic@agr.hr (M.J.); 4 Centre of Excellence for Biodiversity and Molecular Plant Breeding (CroP-BioDiv), 10000 Zagreb, Croatia

**Keywords:** grapevine viruses, indigenous cultivar, leafroll, screening, molecular characterization

## Abstract

The cultivar Plavac Mali (*Vitis vinifera* L.), the most important indigenous red grapevine cultivar in Croatia, was tested for the presence of 16 grapevine viruses. Thirty-five samples from the collection vineyard were tested for the presence of grapevine leafroll-associated viruses-1, -2, and -3 (GLRaV-1, GLRaV-2 and GLRaV-3, respectively), grapevine fanleaf virus (GFLV), arabis mosaic virus (ArMV), grapevine virus-A (GVA), -B (GVB), -G (GVG), -H (GVH), -I (GVI), -J (GVJ), grapevine fleck virus (GFkV), grapevine rupestris stem pitting associated virus (GRSPaV), and grapevine pinot gris virus (GPGV) by reverse transcription–polymerase chain reaction (RT-PCR). Furthermore, standard PCR was conducted for grapevine badnavirus 1 (GBV-1) and grapevine red blotch virus (GRBV). Mixed infections were most common and GLRaV-3, the most abundant virus found in 85.71% of the vines tested, was further molecularly characterised. Different genomic variants of the heat shock protein homologue (HSP70h) were separated by cloning, detected by single-strand conformation polymorphism (SSCP) analysis, sequenced, and phylogenetically analysed. The presence of phylogenetic groups I and II was only confirmed. This study demonstrates the high virus infection rate of Plavac Mali vines and the heterogeneity of GLRaV-3 present nowadays in a collection vineyard.

## 1. Introduction

Plavac Mali (*Vitis vinifera* L.) is the most important Croatian grapevine cultivar used for red wine production and the second rated cultivar in terms of acreages represented [1]. It is mostly grown throughout the central and southern Adriatic coast area. A direct descendant of Tribidrag, also known as Zinfandel or Primitivo [2,3], this cultivar was deeply analysed in terms of ampelographic [4,5], genetic [6], and biochemical characteristics of the berries [7] and wine properties [8,9]. For the rising awareness of the beneficial health aspect of red wine, this cultivar has been gaining more attention over the past years. The wine produced from Plavac Mali is distinguished by higher antioxidant activity, total phenolic content, and catechin concentrations compared with wines from some other native and introduced grapevine cultivars in this region [8]. As a native cultivar, it is highly appreciated for viticultural practice due to its high drought tolerance and resistance to various pathogens [10].

Grapevine viability and performance can be affected by various agents. One of the crucial factors is the presence of viruses and virus-like diseases, which have a negative impact on every aspect of plant life and, potentially, wine quality. Grapevine could be affected by more than 80 viruses and virus-like agents [11]. Those which can trigger severe plant changes are considered more dangerous and are included in the certification programs of many countries. Nepoviruses such as grapevine fanleaf virus (GFLV) and arabis mosaic virus (ArMV) are associated with the disease complex known as infective degeneration, causing malformation of berries, leaves, and canes, reducing the longevity of vines and fruit quality [12]. The main viral agents that cause grapevine leafroll disease (GLD) are potentially so dangerous that GLD is comparable with several fungal diseases [13,14,15]. The disease delays the ripening, alters the sugar concentration, decreases the photosynthetic rate, and influences the overall quality of grapes [16]. The primary etiological agent contributing to this disease is GLRaV-3 [17], although GLD may also be associated with other viral agents from genus *Ampelovirus* (GLRaV-1; [16]) and *Closterovirus* (GLRaV-2). Grapevine fleck virus (GFkV) from genus *Maculavirus* represents a latent threat particularly to rootstock material [18] and therefore is also mandatory in the certification programs.

Other viral agents in some countries are considered less pathogenic to grapevine growing and are mandatory in certification programs of planting material depending on the country. These are, for example, grapevine rupestris stem pitting associated virus (GRSPaV, [19]) and grapevine viruses A and B (GVA, GVB; [19]), viral agents linked to rugose wood disease. Nouvelle members of the genus *Vitivirus* associated with this disease are emerging from different wine-growing regions around the world [20], and some of them pose a real threat for grapevine growth, especially in mixed infections [21]. Likewise, the grapevine Pinot gris virus (GPGV), a *Trichovirus,* has recently come into focus for its harmful effects on grapevine plants [22]. With this point considered, more extensive testing for these viruses could become even more critical for obtaining high-quality planting material.

Even though grapevine virome is mostly consisted of RNA viruses, in recent years, by implementing high-throughput sequencing (HTS) in plant virome research, several DNA viruses were detected in grapevine [23]. One of them, grapevine badnavirus 1 (GBV-1), was so far only found in Croatia in two indigenous varieties, Ljutun and Vlaška [24].

Identifying the individual variants which constitute virus inoculum and expanding the knowledge on their interaction with the host plant further contribute to efforts to control and monitor this virus. Since the first complete study was undertaken by Turturo et al. [25] on the GLRaV-3 population structure, new divergent isolates from around the globe have come to attention [26,27]. Eight major GLRaV-3 phylogenetic groups have been identified so far [28]. As a positive-sense single-stranded RNA virus, GLRaV-3 is predisposed to successive generations of different genetic variants, forming together complex populations [29]. The genome of GLRaV-3 is divided into 12 ORFs [17], with HSP70h (ORF 4) being one of the regions commonly used for molecular characterization [16,25,28]. ORF 4-encoded homologue protein is also well known to facilitate cell-to-cell movement and the formation of the virion head, both of which are important for disease development [16,17]. So far, a clear link between the population structure of GLRaV-3 infection and the biological response of the host plant was not established [16]. After infecting grapevine hosts, GLRaV-3 takes permanent residence [16]; therefore, an old and widespread cultivar Plavac Mali, which has great potential for added-value premium wines and sustainable viticulture, represents a valuable material to study viral distribution with a much closer insight into the genetic diversity of GLRaV-3. Studies conducted so far on the sanitary status of Plavac Mali were mostly based on serological methods [30] and, to a lesser extent, molecular methods [31] with limited information on GLRaV-3’s genetic diversity. The biological response of Plavac Mali has also not been evaluated with respect to a specific GLRaV-3 variant and other viruses.

In this study, using sensitive and reliable PCR-based detection methods, the presence of 16 economically important viruses was tested, and analysis of GLRaV-3 genetic variants was undertaken. This study provides valuable data on the sanitary status of Croatia’s most important red-berried cultivar and represents an important prerequisite for the production of healthy certified grapevine planting material.

## 2. Results

**Figure 1:**
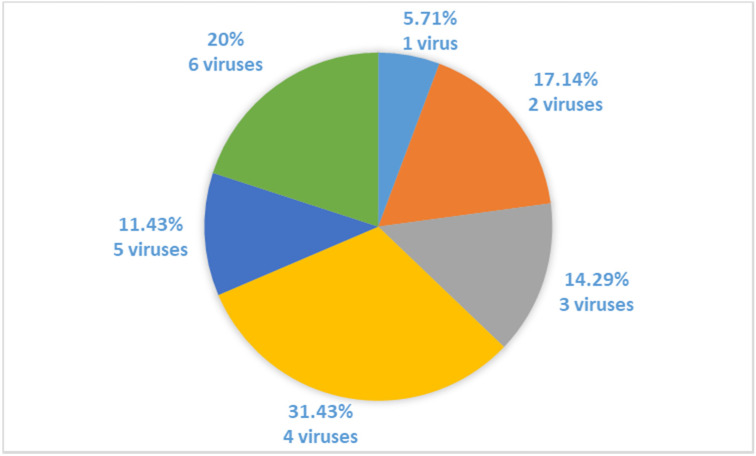
Percentages of plants infected with a different number of viruses in analysed vines of cv. Plavac Mali.

Molecular detection using primers specific for 16 grapevine viruses confirmed that neither one of 35 analysed plants of Croatian indigenous cultivar Plavac Mali was virus-free. Plants infected with four and six viruses represent the majority of the plant population, as four viruses were detected in 31.43% and six viruses in 20% of samples tested. Only 5.71% of analysed plants were infected with only one virus (Figure 1).

**Figure 2:**
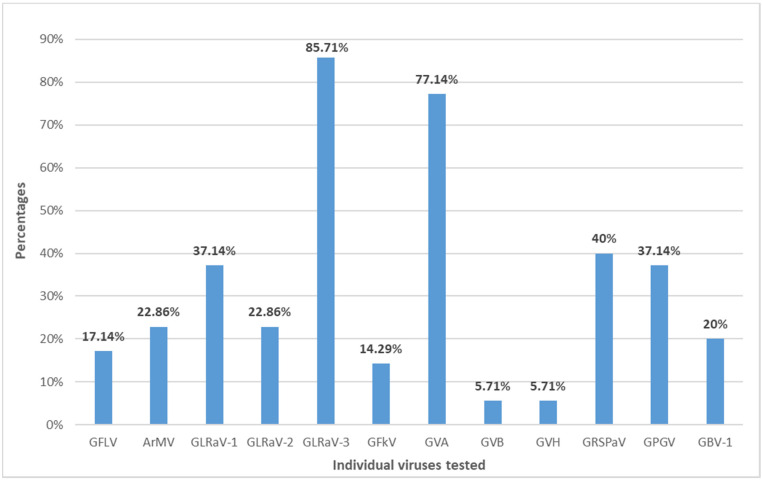
The percentage of c.v. Plavac Mali plants infected with different grapevine viruses.

The highest virus prevalence was reported for GLRaV-3, detected in 85.71% of samples. The GVA was detected in 77.14%, and GRSPaV was detected in 40% of tested samples. The lowest prevalence was observed for GVB and GVH, which were detected in 5.71% of the tested samples, while the prevalence of the other viruses detected in the sample ranged from 14.29% to 37.14% (Figure 2). The GVG, GVI, GVJ, and GRBV were not detected in tested samples.

**Figure 3:**
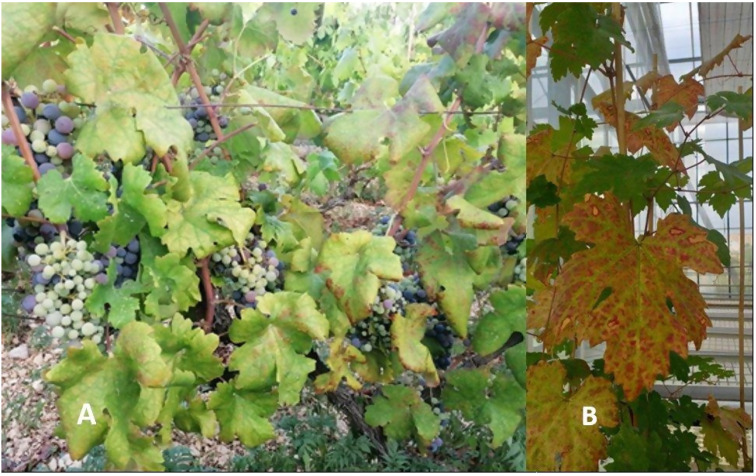
Symptoms of grapevine leafroll disease on two clones of cv. Plavac Mali, one harbouring multiple infections with GLRaV-1, GVA, GLRaV-3 and GRSPaV in a field-grown grapevine displaying moderate symptoms (clone TG; **A**), and the other plant (clone 217/1) tested positive for GLRaV-3 only and grown in greenhouse conditions displaying moderate symptoms (**B**).

Regardless of the high virus prevalence and frequent mixed infections, the symptoms of grapevine leafroll disease observed in the field plants were not unique, and they could not be associated with particular virus composition. In 2020 most of the symptoms observed on individual plants were ranked as moderate (34.29%), which presumes that more than 50% of leaves of the tested plants were diagnosed with interveinal reddening and downroll, the main characteristic of GLD (Table 1). These changes were observed in the late summer (August and September). Asymptomatic plants (31.43%) were also represented in a significant number. Some plants (11.43%) have developed the most severe symptoms, such as retarded growth or complete decline in 2020, which resulted in plant death the following year (Table 1). In contrast to 2020, among surviving plants in 2021, mostly mild symptoms were observed (69%), and no plants were ranked as severe, with only two plants harbouring moderate symptoms. Some of the symptoms observed are presented in Figure 3.

Nine plants originating from nine scattered wine-growing areas were further selected based on the symptom severity observed in the first year of assessment for the molecular characterization of the HSP70h region of GLRaV-3. Clones 020 and 268 expressed severe symptoms, clones 214, 036, and 096 moderate, clones 041 and 225 mild, while clones 201 and 008 displayed no symptoms.

**Figure 4:**
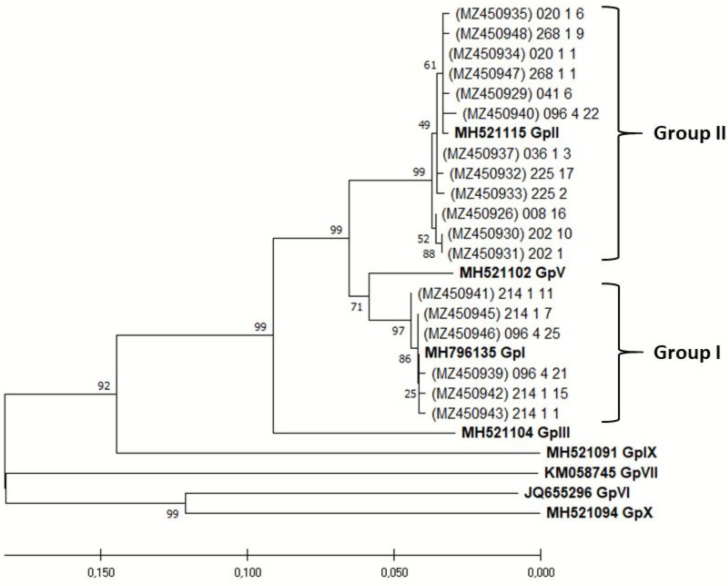
Neighbourhood-joining tree with bootstrap values gained by analysing HSP70h sequence of GLRaV-3 isolates from tested plants and their accession numbers. Referent groups, which are bolded, marked as GpI, GpII, GpIII, GpV, GpVI, GpVII, GpIX and GpX were according to Diaz-Lara et al. [28]. Clusters were determined in accordance with referent groups. Phylogenetic analysis was performed in MEGA 5.

The SSCP analysis was performed on approximately 20 randomly selected bacterial colonies per sample (nine selected plants). In total, 177 virus variants were analysed, and all variants of each sample displaying different SSCP patterns were sequenced. The phylogenetic analysis revealed that most of them (69.66%) belong to GLRav-3 phylogenetic group II, while 30.43% of variants belong to phylogenetic group I, all supported by high bootstrap values (Figure 4.). When analyzing sample virus population heterogeneity, 44.44% of samples had uniform virus populations consisting of one variant (one SSCP pattern) only. In the majority of samples (55.56%), the clear predominance (>60%) of one variant (SSCP-pattern) was reported. In only 11.11% of these samples, detected variants belonged to different phylogenetic groups. Mean genetic diversity for the entire population was 0.029 ± 0.005, while it was 0.002 ± 0.001 for sequences belonging to phylogenetic group I and 0.012 ± 0.002 for phylogenetic group II.

## 3. Discussion

Grapevine viruses and viral diseases have a long history of scientific investigation in Croatia. Since the first report on the presence of the infection degeneration complex in Croatia by Šarić and Corte [32], continuous investigations for the presence of viruses have been carried out in all viticultural regions of Croatia. The results have consistently indicated a high infection rate, especially in vineyards along the central and southern Adriatic coast, where the most appreciated cultivar for viticulture is Plavac Mali [5,30].

Although most of the studies conducted so far are based on serological ELISA testing [30,33,34], some recent studies have introduced advanced molecular methods in studying viral populations on indigenous grapevine cultivars of the Mediterranean region of Croatia [31,35]. In using the PCR-based methods, molecular cloning, and sequencing, this study is thus far the most comprehensive, conducted to determine the genetic variants of the most predominant virus, GLRaV-3, based on HSP70h sequence variants obtained from isolates of cv. Plavac Mali. Originated from different wine-growing locations and introduced into the collection with initial sanitary status [5], each clone could have been exposed to infections by vector transmission. Viral composition within the plant is not a permanent state, and with the sufficient time lag, it could potentially be used for estimating virus spread through the vineyard. We confirmed deteriorated sanitary status of the cv. Plavac Mali with GLRaV-3 as the most common virus detected in 85.71% of tested plants.

We assume that the horizontal transmission of the viruses between clonal candidates has been occurring since the collection establishment in 2005, even though visual inspection gave negative results regarding *Coccidae* and *Pseudococcidae*. Zdunić et al. [5] reported GLRaV-3 prevalence similar to the one reported in our study, indicating GLRaV-3 poor transmission within this collection vineyard. Although slow in spreading in such a system, its high prevalence poses a significant threat to the production of high-quality red wines due to known GLRaV-3 impact on grape ripening and berry metabolism. Consequently, sugar and polyphenol content could be significantly reduced [36,37]. According to field symptoms in our study, the expression of symptoms in this cultivar varies from asymptomatic to severely deteriorated plants, depending also on the year observed for each individual clone (Table 1). Out of the most apparent symptoms related to GLRaV-3, interveinal reddening and downrolling were observed. However, GLRaV-3 was just one of the agents in multiple virus infections, and since only two plants infected exclusively with GLRaV-3 were found in the collection (Table 1), the symptomatology was challenging to interpret in relation to a particular virus; thus, no correlation was observed between GLRaV-3 variants and symptom expression in the field plants. The same clone, infected only with GLRaV-3, was propagated by cuttings and grown in controlled greenhouse conditions and contrary to field plants symptom expression in the first year observed displayed moderate GLD symptoms (Table 1). Following this example and the difference in symptom expression between the two years observed, we can conclude that environmental conditions have an impact on symptoms expression. The occurrence of other tested GLD agents, GLRaV-1 (37.14%), and GLRaV-2 (22.85%), further adds to the difficulty of symptom assessment in the field, with possibly even higher occurrence of GLRaV-1, since primers used in this study could have had difficulties to detect specific genetic variants of this particular virus [38]. 

Furthermore, clones infected with numerous viruses, such as clone 020 infected with six different viruses, developed severe virus symptoms, resulting in the host plant decline and death. With no physiology analyses of those plants of cv. Plavac Mali, we can only assume that besides observed symptomatology, virus composition influenced plant metabolism and potentially wine quality. Hančević et al. [39] showed that growth proliferation of in vitro cultivated Plavac Mali proved to be highly virus composition dependent. In this study, a very high incidence of infection, as well as multiple virus infection, was found in the population of Plavac Mali field plants (Table 1). One of the ampelographic traits of cv. Plavac Mali is a large number of green berries in a ripe cluster [5,7], also found here while inspecting the field symptoms, so it would be intriguing to verify if this trait is caused by any of viral agents or by cultivar itself in interaction with some environmental factors and viticultural practice.

According to the HSP70h, phylogenetic groups I and II were detected in this study, showing relatively low genetic diversity of GLRaV-3 based on SSCP method and sequence variants, similarly as in other native vines from the Mediterranean part of Croatia found in the same collection [35]. Monophyletic infections prevailed over polyphyletic ones, and in monophyletic infections, Group II was dominant over Group I. Prevalence of monophyletic infections was also reported so far in Spain [40], Portugal [41] and China [42]. Mixed infections of two monophyletic variants were found in 11.11% of samples, which is lower than the average value of the other indigenous cultivars of the Mediterranean part of Croatia found by previous studies (18% found by Hančević et al. [35]). The HSP70h primers used in that study were designed in 2005 [25] and displayed some limitations in detecting subsequently identified isolates from phylogenetic groups VI and X [27]. However, in our study, no cases of inefficient amplification were observed. This corresponds to the work done by Hančević et al. [35] on indigenous cultivars of the Mediterranean part of Croatia where the ELISA method was used, along with the primers in multiplex test [43], and no cases were observed where ELISA positive sample tested negative by RT-PCR. Even so, with limitations of primers taken into consideration, we cannot exclude the possible presence of other GLRaV-3 isolates in mixed infections with detected ones.

Prevalence of GVA (77.14%) was slightly higher than previously suggested by other authors [34], and the high occurrence of GRSPaV in our study (40%) confirmed the wide presence of GRSPaV in vineyards in the Mediterranean basin [44], although to a lesser extent than in other similar surveys in Croatia [31]. The reason for this could be in initial wine-growing locations from which individual vines for this study originated (Table 1). They represent some of the most valuable terroirs for cultivating Plavac Mali, but to some extent are isolated locations such as islands Brač, Hvar, and Vis, with a low exchange of plant material. Another member of the genus *Vitivirus*, GVH, was detected in 5.71% of samples originating from only one location (Table 1). Its worldwide distribution is still relatively unknown, considering it was first discovered in Portugal in 2018. [45], followed by its mention in several cultivars in the U.S. Department of Agriculture National Clonal Germplasm Repository in Winters [20] and recently in the cultivar Assyrtiko in Greece [46]. In Croatia, it has been recorded by the National collection of Croatian autochthonous grapevine cultivars located in the experimental station “Jazbina” in nine different cultivars with an infection rate of 7.9% [47].

The prevalence of nepoviruses (ArMV 22.86% and GFLV 17.14%) were lower than suggested by the previous study of cv. Plavac Mali [30] and higher than other indigenous cultivars of this area [35]. Using ELISA analysed samples from the same collection, Zdunić et al. [5] recorded only 6% ArMV positive plants, indicating the presence of vectors and natural spread of nepoviruses within the collection vineyard. Another explanation for such a difference in nepoviruses presence is a method used for detection purposes. Observed chlorotic yellowing on leaves in the springtime and reduced berry size at the time of harvest could be associated with nepoviruses infection.

A possible reason for the high occurrence of GPGV (37.14%) is the presence of *Colomerus vitis* (Pagenstecher), a mite described as a vector for GPGV [48], visually confirmed in the collection vineyard. This virus originates from Asia [49] and is considered to be brought into Croatia by importing rootstock material. Although significant effects on grapevine yield were reported [50], no specific GPGV symptoms of deformation and leaf mottling were observed in cv. Plavac mali.

Grapevine badnavirus 1, a member of the family *Caulimoviridae*, is a nouvelle grapevine virus which, to our knowledge, has been found only in the Croatian autochthonous grapevine cultivars Ljutun and Vlaška, whose origin is also in the Mediterranean part of Croatia [24]. Testing for this particular virus was performed with three different sets. Sample 026/1, which tested positive with two sets of primers (F6/R6, F13/R13) but not with the F2/R2 primer set, possibly indicates a higher level of genetic variability of this particular virus (Supplementary Figure S1).

## 4. Materials and Methods

### 4.1. Plant Material and Symptoms Assessment

The plant material was analysed from the collection vineyard of Plavac Mali located at the Institute for Adriatic Crops and Karst Reclamation [5] that encompasses all divergent clonal candidates of Plavac Mali grafted on certified rootstock Borner (*Vitis riparia* 183G × *Vitis cinerea* Arnold). Clones included in this study were selected based on the presence of visual symptoms of viral infections, and in total, thirty-five samples originating from 13 different wine-growing locations were inspected and assayed (Table 1). Grapevine leafroll disease symptoms were assessed in 2020 and 2021 throughout the summer period and into the early autumn and classified as asymptomatic, mild symptoms (occasional interveinal reddening possibly associated with GLD), moderate (interveinal reddening with leaf downroll that affected up to half of the plant leaves), and severe (extensive interveinal reddening with leaf downroll with more than one- half of the leaves affected).

### 4.2. RNA Extraction, cDNA Synthesis and DNA Extraction

For detecting GFLV, ArMV, GLRaV-1, -2, -3, GVA, GVB, GFkV, GRSPaV and GPGV dormant canes were collected in the winter period (December). Cortical scrapings were homogenized (250 mg of tissue) using liquid nitrogen, and RNA extraction was performed as described by MacKenzie et al. [51] using RNeasy Plant mini kit (Qiagen, Hilden, Germany). cDNA synthesis was performed in the total volume of 22 µL, using 12 µL of the RNA template, 200 U of M-MLV reverse transcriptase (Invitrogen, Waltham, MA, USA), 100 U of RNase inhibitor (Invitrogen, Waltham, MA, USA), 0.1 M DTT (Invitrogen, Waltham, MA, USA), 0.5 mM dNTP mix and 50 µM random nonamers (Sigma Aldrich, St. Louis, MO, USA). For the detection of vitiviruses (GVG, GVH, GVI, and GVJ), RNA was isolated from petioles using the RNeasy Plant Mini Kit (Qiagen, Hilden, Germany), while for grapevine red blotch virus (GRBV) and grapevine badnavirus 1 (GBV-1), DNA was isolated from the same tissue using the DNeasy Plant Mini Kit (Qiagen, Hilden, Germany), both according to the manufacturer’s instructions. The quality and quantity of isolated DNA/RNA were checked using NanoPhotometer P330 spectrophotometer (Implen, München, Germany).

### 4.3. Virus Detection

Detection of grapevine leafroll associated viruses-1, -2, and -3, (GLRaV-1, -2, and -3), grapevine virus A (GVA), grapevine virus B (GVB), grapevine rupestris stem pitting associated virus (GRSPaV), grapevine fanleaf virus (GFLV), arabis mosaic virus (ArMV), and grapevine fleck virus (GFkV) was performed by multiplex RT-PCR reaction as described by Gambino and Gribaudo [43]. Primers amplifying the host’s 18S rRNA fragment were used as an internal control. Amplicons were detected electrophoretically according to their expected size as follows: 18S rRNA (844bp), GPGV (588bp) GLRaV-2 (543bp), GVB (460bp), ArMV (402bp), GLRaV-3 (336bp), GVA (272bp), GLRAV-1 (232bp), GFkV (179bp), GRSPaV (155bp), and GFLV (118bp.)

Grapevine Pinot gris virus (GPGV) detection was performed separately using PCR conditions by Saldarelli et al. [22] and primers as described by Morelli et al. [52].

Detection of GVG, GVH, GVI and GVJ was performed with the One-step RT-PCR kit (Qiagen, Hilden, Germany) using primers described by Diaz Lara et al. [20]. The master mix consisted of 0.4 µM of each primer, 5–10 ng of the RNA template, and other components recommended by the manufacturer. RT-PCR was performed on Mastercycler (Eppendorf, Hamburg, Germany) under the following conditions: reverse transcription 50 °C/30 min, initial PCR activation step 95 °C/15 min, 35 cycles: denaturation 94 °C/30 s, annealing 52 °C/30 s, extension 72 °C/1 min, and final extension 72 °C/10 min.

Detection of GRBV was performed with primers GVGF1/GVGR1 as described by Al Rwahnih et al. [53], while detection of GBV-1 was performed with primer pairs F2/R2, F6/R6, and F13/R13 as described by Vončina and Almeida [24]. The master mix was prepared with HotStartTaq DNA Polymerase (Qiagen, Hilden, Germany) using 0.4 µM of each primer and all other components according to the manufacturer’s instructions. PCR was performed on the same instrument under the same conditions described above but without the reverse transcription step.

### 4.4. Cloning and Single-Stranded Conformational Polymorphism (SSCP) Analysis

All GLRaV-3 positive samples were further analysed to determine their genetic variability. To obtain the longer product, primers (LC1 and LC2) amplifying the 545 bp long region of the HSP70h gene were used [25]. The reaction conditions were as described by the same author, with the exception of 15 min prolonged final extension for enhancing cloning efficiency.

To separate different genomic variants from each sample, amplicons were TA-cloned using pGEM-T Easy Vector Sistem I (Promega, Madison, WI, USA) and ligation products were used to transform *E. coli* JM109-High-Efficiency Competence cells (Promega, Madison, WI, USA). The presence of the insert was confirmed by the white–blue selection, and at least 20 transformed colonies per sample were further characterised.

To identify separated variants by SSCP, inserts originating from selected colonies were amplified using the same primer set (LC1/LC2) as in the initial reaction. 2 µL of amplicons were denatured and analysed after silver staining of polyacrylamide gels as described by Černi et al. [54]. Example of identification of different genomic variants of GLRaV-3 by SSCP analysis is displayed in Supplementary Figure S2.

### 4.5. Sequencing and Sequence Analysis

After identifying different sequence variants, at least two of them displaying the same SSCP pattern were sent for sequencing with Macrogen Europe Inc. (Amsterdam, The Netherlands). Representative sequences were deposited in GeneBank under the accession numbers: MZ450926-MZ450948. Sequences obtained were aligned in Clustal X 2.1 [55] and analysed in Mega 5 [56]. The phylogenetic tree was obtained using the neighbourhood-joining method and Tamura–Nei evolutionary model. Bootstrap analysis was based on 1000 repetitions, and reference sequences for different GLRaV-3 phylogenetic groups were obtained from Diaz-Lara et al. [28]. Sequenced bacterial colonies along with their representative phylogenetic cluster as well as the GeneBank accession number are listed in Supplementary Table S1.

## 5. Conclusions

We confirmed the deteriorating sanitary status of the most widespread Croatian indigenous red cultivar Plavac Mali. Mixed infections were common in the tested vines, and none of the tested clones proved to be virus-free. GLRaV-3 was the most common virus detected in 85.71% of the samples. Among its phylogenetic variants according to HSP70h, groups I and II were detected, with the monophyletic type of infections as predominant. Diagnostic tools improvement and knowledge about the sanitary status of grapevine plants with more detailed insight into genetic differences within the GLRaV-3 population should lead to better planning and management strategy for this valued wine cultivar in the Mediterranean Croatia viticulture. Promoting the production and usage of virus-free material should be taken more seriously in the future in order to realize the full potential of Plavac Mali.

## Figures and Tables

**Table 1:** The presence of different grapevine viruses in clones of cv. Plavac Mali originated from different wine-growing areas and the Grapevine leafroll disease symptom assessment on selected clones of cv. Plavac Mali in two consecutive years.

Vine-Growing Area	Clone	* GFLV	ArMV	GLRaV-1	GLRaV-2	GLRaV-3	GFkV	GVA	GVB	GVH	GRSPaV	GPGV	GBV-1	GLD Symptoms 2020.	GLD Symptoms 2021.
Šolta	004/3			+	+				+		+			AS	MI
Čiovo	008/1				+	+		+			+			AS	MO
Hvar	010/1	+		+		+		+						MI	MI
	020/1	+	+			+	+	+					+	S	-
	020/2	+	+			+	+	+					+	S	-
	026/1			+		+		+				+	+	AS	AS
	026/3			+		+		+					+	AS	AS
	027/3							+			+			AS	AS
	027/4					+		+			+			AS	MI
Zemunik	036/1				+	+		+		+	+	+		MO	MI
	036/3				+	+		+		+				MO	MI
Svinišće	041/1				+	+		+				+		MI	MI
Labin	043/1			+	+	+			+		+	+		MO	AS
Pelješac	056/1			+				+						MI	AS
	059/3	+		+		+		+			+			MO	-
Korčula	091/1					+		+				+		MO	MI
	095/1						+					+		AS	AS
	096/4	+		+		+		+						MO	MI
	096/5			+							+			MO	MI
Vrgorac	202/3					+		+			+			AS	MI
Vis	210/1		+	+		+		+						MO	MI
	210/5					+		+						MO	MI
	214/1		+			+	+	+				+	+	MO	MI
	214/2		+			+	+	+				+	+	MO	MI
	215/1		+			+		+				+		MI	MI
	215/3		+		+	+		+				+		MI	MI
	217/1					+								AS	MI
	217/2					+								AS	MI
	217/3					+						+		AS	MI
	217/4					+		+			+			MI	MI
	217/5	+				+					+	+		MI	MI
Kaštela	225/3				+	+		+			+	+		MI	MI
Brač	268/1		+	+		+		+			+		+	S	AS
	268/2			+		+		+						S	AS
Trogir	TG			+		+		+			+			MO	MO

* Abbreviations represent the grapevine viruses as follows: grapevine fanleaf virus, arabis mosaic virus, grapevine leafroll associated virus-1, -2 and -3, grapevine fleck virus, grapevine virus-A, -B and -H, grapevine ruprestris stem pitting associated virus, grapevine Pinot gris virus and grapevine badnavirus 1. Grapevine leafroll disease symptoms (GLD symptoms) are ranked as asymptomatic (AS), mild (MI), moderate (MO) and severe (S). Test results for grapevine virus-G, -I, -J and Grapevine red blotch virus (GRBV) are not presented in this table because all samples tested negative.

## Data Availability

Representative sequences are deposited in GeneBank under the accession numbers: MZ450926-MZ450948.

## Supplementary Materials

The following supporting information can be downloaded at: https://www.mdpi.com/article/10.3390/pathogens11020176/s1, Figure S1: An example of gel electrophoresis of PCR products obtained for grapevine badnavirus 1 using the HotStartTaq DNA Polymerase Kit (Qiagen, Hilden, Germany) and different primer combinations targeting different viral genome regions (F2/R2—product size 731 base pairs (bp), F6/R6—850 bp and F13/R13—534 bp). Lines: M—GelPilot 100 bp Plus Ladder (Qiagen, Hilden, Germany), P—positive control, 1—020/1, 2—020/2, 3—026/1, 4—026/3, 5—214/1, 6—214/2, 7—261/1, N—negative control. Figure S2: The identification of different genomic variants of GLRaV-3 by single-strand conformation polymorphism (SSCP) analysis. Clones displaying pattern A have the same nucleotide composition, while clone displaying pattern B belongs to different genomic variant, as confirmed by sequencing. Table S1: title: Sequenced bacterial colonies obtained from individual vines along with their accession number and phylogenetic cluster they represent.
