# Supplementary material for: Prevalence of Virus Infections and GLRaV-3 Genetic Diversity in Selected Clones of Croatian Indigenous Grapevine Cultivar Plavac Mali
Source: Pathogens. 2022 Jan 27;11(2):176. doi: 10.3390/pathogens11020176 (PMC8876015; doi:10.3390/pathogens11020176)
Supplement: Supplementary file 1 [file pathogens-11-00176-s001.zip › pathogens-1529975-supplementary.pdf]

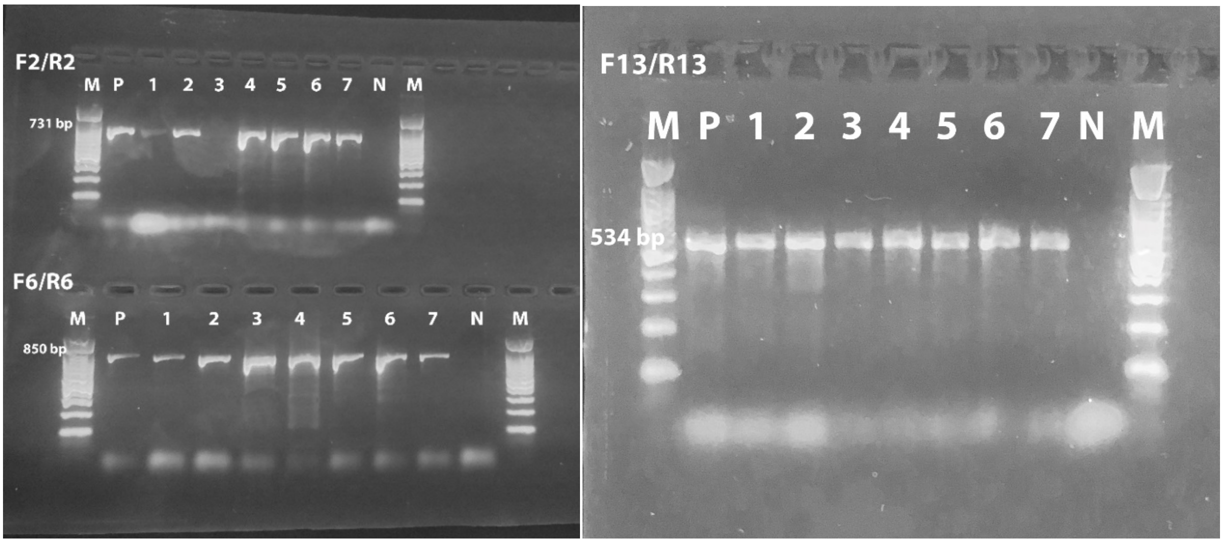

Supplementary Figure S1. An example of gel electrophoresis of PCR products obtained for grapevine badnavirus 1 using the HotStartTaq DNA Polymerase Kit (Qiagen, Hilden, Germany) and different primer combinations targeting different viral genome re-gions (F2/R2 - product size 731 base pairs (bp), F6/R6 - 850 bp and F13/R13 - 534 bp). Lines: M - GelPilot 100 bp Plus Ladder (Qiagen, Hilden, Germany), P - positive control, 1 - 020/1, 2 – 020/2, 3 - 026/1, 4 - 026/3, 5 - 214/1, 6 - 214/2, 7 - 261/1, N - negative control.

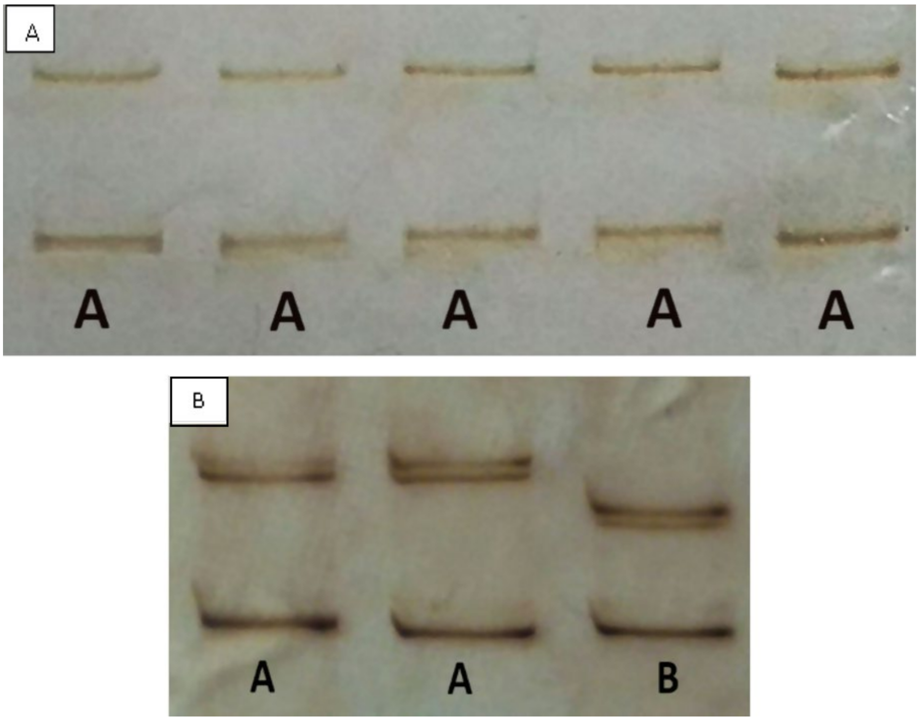

Supplementary Figure S2. The identification of different genomic variants of GLRaV-3 by single-strand conformation polymorphism (SSCP) analysis. Clones displaying pattern A have the same nucleotide composition, while clone displaying pattern B belongs to different genomic variant, as confirmed by sequencing.

Supplementary Table S1. Sequenced bacterial colonies obtained from individual vines along with their accession number and phylogenetic cluster they represent

Clone_bacterial colony	Accession number	Cluster
008_16	MZ450926	II
008_26	MZ450927	II
041_30	MZ450928	II
041_6	MZ450929	II
202_10	MZ450930	II
202_1	MZ450931	II
225_17	MZ450932	II
225_2	MZ450933	II
020_1_1	MZ450934	II
020_1_6	MZ450935	II
036_1_14	MZ450936	II
036_1_3	MZ450937	II
036_1_7	MZ450938	II
096_4_21	MZ450939	I
096_4_22	MZ450940	II
214_1_11	MZ450941	I
214_1_15	MZ450942	I
214_1_1	MZ450943	I
214_1_6	MZ450944	I
214_1_7	MZ450945	I
096_4_25	MZ450946	I
268_1_1	MZ450947	II
268_1_9	MZ450948	II
